# P-560. The Genomic Epidemiology of Fulminant Streptococcus pyogenes Infections in Rochester, NY: Why Community Surveillance Matters

**DOI:** 10.1093/ofid/ofaf695.775

**Published:** 2026-01-11

**Authors:** Vishal Fnu, Francois Lebreton, Ting Luo, Yoon Kwak, Jason Bennett, Patrick McGann, Emil P Lesho

**Affiliations:** Rochester Regional Health, Rochester, VA; Walter Reed Army Institute of Research, Silver Spring, Maryland; Walter Reed Army Institute of Research, Silver Spring, Maryland; Walter Reed Army Institute of Research, Silver Spring, Maryland; Walter Reed Army Institute of Research, Silver Spring, Maryland; Walter Reed Army Institute of Research, Silver Spring, Maryland; Rochester Regional Health, Rochester, VA

## Abstract

**Background:**

Group A *Streptococcus pyogenes* (GAS) is a leading cause of infectious death globally. Hospital acquired (HA) GAS is particularly burdensome for infection preventionists, administrators, and involved healthcare workers (HCW). After a cluster of fulminant infections (death < / = 48 hrs. from symptom onset), we sought to evaluate the genetic relatedness of the isolates to an HA GAS outbreak that occurred at our facility in 2017and ascertain the genomic epidemiology of invasive GAS (iGAS).

**Figure:**
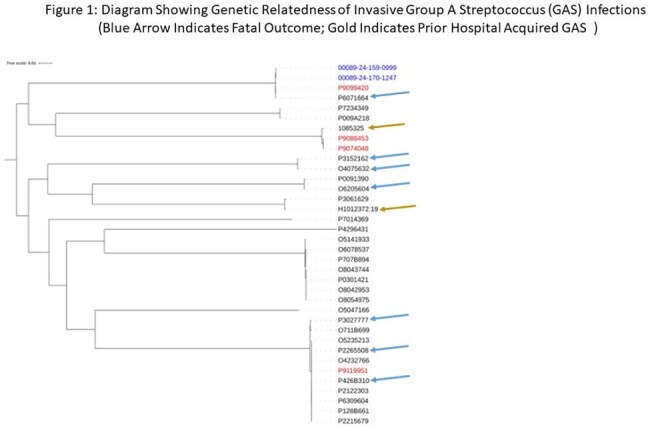

**Figure 2:**
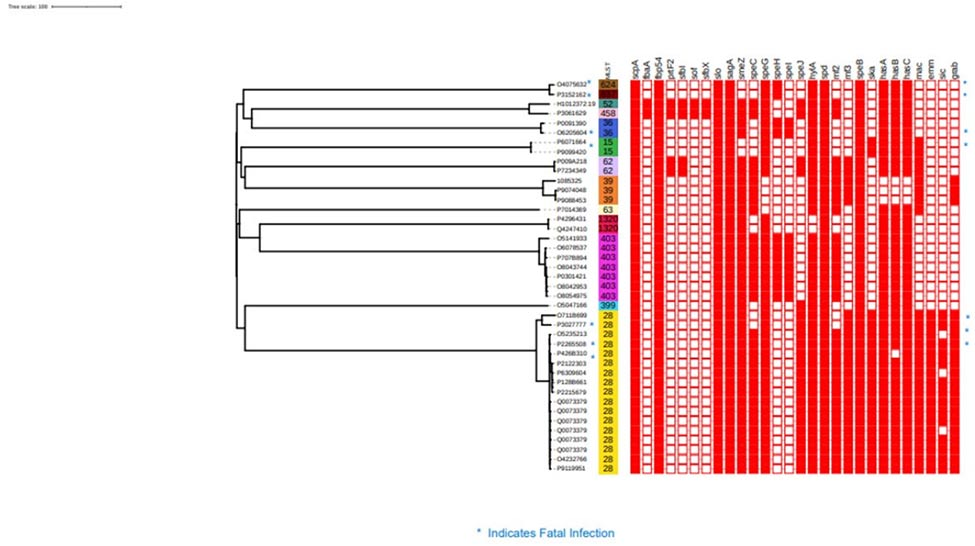
Heat map showing virulence gene content of 36 Group A Streptococcus isolates

**Methods:**

Contact tracing and testing of all HWCs with close contact to any patient with possible HA iGAS and an ambidirectional approach were used. All iGAS one year preceding and one year following the index case were collected and underwent whole genome sequencing (WGS). Medical records of the cases prior and subsequent to the index case were retro- and prospectively reviewed. Additionally, six individual GAS colonies from the throat culture of the only positive HCW were sequenced to assess for intra-colony variation. Results were stratified according to outcome, strain type (ST), and *emm* type.

**Table 1:**
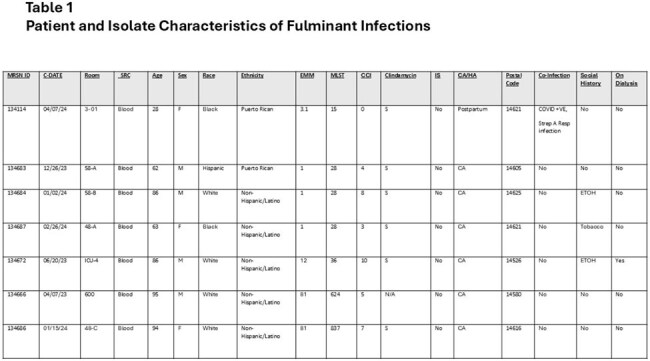
Patient and Isolate Characteristics of Fulminant Infections

**Results:**

From 01/01/2023 to 04/01/2025, 40 isolates from 36 patients and the only positive HCW clustered into 13 lineages and 12 *emm* types. Within each lineage, clusters of high genetic relatedness were identified. However, all isolates from patients with any spatial-temporal or other epidemiologic connection were unrelated, including the ones from the 2017 outbreak (Figure 1). The index isolate belonged to ST-15 emm 3.1. Virulence gene content visually correlated with ST (Figure 2). Although some of isolates from fulminant infections were genetically related, there was no spatial, temporal, or epidemiologic connections among those isolates (Figure 1). There was no association between outcome and ST, or virulence gene content, or antibiotic resistance gene content (Figure 2, Table 1).

**Conclusion:**

All iGAS infections, including all fulminant infections, were community acquired. We found no citation explicitly reporting a ST 15 *emm* sub-type 3.1 in the U.S. Given the elusiveness of iGAS source attribution, these findings illustrate the importance of bolstering community surveillance efforts so that community data can inform outbreak investigation of HA GAS.

**Disclosures:**

All Authors: No reported disclosures

